# Human umbilical cord Wharton jelly cells promote extra-pancreatic insulin formation and repair of renal damage in STZ-induced diabetic mice

**DOI:** 10.1186/s12964-017-0199-5

**Published:** 2017-10-17

**Authors:** Martin Maldonado, Tianhua Huang, Lujun Yang, Lan Xu, Lian Ma

**Affiliations:** 1 Department of Pediatrics, Second Affiliated Hospital of Shantou University Medical College, Shantou, Guangdong 515041 People’s Republic of China; 2 Translational Medical Center, Second Affiliated Hospital of Shantou University Medical College, 22 Xinling road, Shantou, Guangdong 515041 People’s Republic of China; 3 Guangdong Provincial Key Laboratory of Infectious Diseases and Molecular Immunopathology, Research Center for Reproductive Medicine, Shantou University Medical College, Shantou, 515041 People’s Republic of China; 4 Department of Burns and Plastic Surgery, Second Affiliated Hospital of Shantou University Medical College, Shantou, Guangdong 515041 People’s Republic of China; 5 Reproductive Medicine & Genetics, Chengdu Jinjiang Hospital for Maternal & Child Health Care, Chengdu, 610066 China; 6 Department of Pediatrics, Maternal and Child Health Care Hospital of Shenzhen University, 518052 Shenzhen, Guangdong People’s Republic of China; 7 Department of Pediatrics, Maternal and Child Health Care Hospital of Pingshan District, 518122 Shenzhen, Guangdong People’s Republic of China

**Keywords:** Human umbilical cord Wharton jelly cells, Diabetes type-1, Streptozotocin, Intraperitoneal administration, Insulin

## Abstract

**Background:**

We evaluated the therapeutic effect and fate of high doses of human umbilical cord Wharton jelly cells (hUCWJCs) after IP administration to streptozotocin (STZ)-induced diabetic mice.

**Methods:**

Type 1 diabetes (T1D) was induced in Kunming mice via IP injection of STZ. hUCWJCs were labeled with 1,1′-dioctadecyl-3,3,3′,3′-tetramethylindocarbocyanine perchlorate (DiI). Diabetic animals with sustained hyperglycemia for at least 2 weeks were administered 1 × 10^7^ Dil-hUCWJCs via intraperitoneal injection. Insulin, glucagon and PDX-1 were detected by immunofluorescence with confocal microscopy. Serum mouse and human C-peptide was assayed in blood collected via intracardiac puncture. Specific β-cell differentiation markers and human DNA were assessed using qPCR performed with 200 ng of target DNA.

**Results:**

hUCWJCs migrated to the STZ-damaged organs and contributed to lower blood glucose levels in 30% of the treated mice. Confocal microscopy revealed the presence of resident insulin-positive cells in the liver and kidneys. hUCWJC-treated mice with restored hyperglycemia also showed increased serum mouse C-peptide levels. The qPCR results, particularly in the liver, revealed that after transplantation hUCWJCs upregulated genes of endocrine precursors but failed to express endocrine stage markers. Mice with restored hyperglycemia had reduced urinary volume and lacked glomerular hypertrophy, exhibiting a morphology resembling that of normal glomeruli. Moreover, we also verified that one of the possible mechanisms by which hUCWJCs exert immunosuppressive effects is through down-regulation of the cell surface receptor HLA-1.

**Conclusions:**

We confirmed the potential of IP administration of hUCWJCs and the capability of these cells to migrate to damaged tissues and promote insulin secretion from non-pancreatic local cells and to improve renal damage. These findings confer unique therapeutic properties to hUCWJCs, suggesting a promising future in the treatment of diabetes mellitus.

**Electronic supplementary material:**

The online version of this article (10.1186/s12964-017-0199-5) contains supplementary material, which is available to authorized users.

## Background

Type 1 diabetes (T1D) is an autoimmune disease characterized by T-cell–mediated destruction of insulin-producing β–cells [29]. The reversal of T1D involves whole pancreas or islet cell transplantation in association with nonspecific immunosuppressive therapy [38].

Immunosuppressive therapy not only results in compromised immune function but has the potential for additional complications with long-term use. Due to the mentioned limitations, the use of embryonic stem cells (ESCs), cord blood–derived SCs, adult SCs, bone marrow, genetically engineered cells and mesenchymal stem cells (MSCs) among others are under exhaustive testing with the aim of reversing hyperglycemia [1, 3, 19, 22, 46, 56–58].

Among the different types of MSCs, Human Umbilical Cord Wharton Jelly Cells (hUCWJCs) appear to offer the best clinical advantage due to their unique beneficial characteristics [5].

hUCWJCs are located in a specific mucous proteoglycan-rich matrix known as Wharton’s jelly, which is present in the umbilical cord and derived from the extra-embryonic mesoderm. hUCWJCs share an early developmental origin, multi-lineage differentiation potential and exhibit much more proliferative, immunosuppressive, and even therapeutically active properties than SCs isolated from older adult tissue sources, such as bone marrow or adipose tissue [31].

hUCWJCs attenuate wound inflammation, reprogram resident cells to favor tissue regeneration and inhibit fibrosis [24]. They interact with host cells and influence the SC niche through differentiation and/or paracrine signaling mechanisms [20, 35].

Scientific evidence has indicated that the secretion of trophic, soluble or immunomodulatory factors, known as paracrine signaling factors, may represent the most pivotal underlying mechanism of the effect of MSCs [20, 30, 43].

Moreover, hUCWJCs are poor antigen-presenting cells; do not express MHC class II or the co-stimulatory molecules CD40, CD40L, CD80 and CD86 [36, 52]; and are not prone to undergo malignant transformation [4, 50].

Therefore, in this work, high doses of hUCWJCs were used to evaluate the potential of intraperitoneal (IP) administration to improve hyperglycemia symptoms that could rescue STZ-treated mice. We also aimed to analyze their homing properties as well as the mechanisms by which hUCWJCs promote insulin formation and improve renal damage that is typical of patients with diabetes mellitus.

## Methods

### Animals

Male Kunming mice were maintained in a specific pathogen-free animal facility in individual ventilated cages and housed at a temperature of 23/25 °C under a 12-h dark/light cycle. Water and food were given ad libitum*.* All animal experiments were performed using protocols approved by the Institutional Review Board of Shantou University Medical College.

Mice were monitored daily by laboratory members and by animal health technicians. Prior to the experimental endpoint, mice experienced minimal pain or stress during routine handling, body weight determination, and blood collection from the tail vein to measure blood glucose levels.

No ill or deceased mice were observed prior to the experimental endpoint. Mice were euthanized by the cervical dislocation technique.

### Generation of the type 1 diabetic mouse model

STZ was administered IP to selectively destroy pancreatic β-cells and induce a condition resembling T1D. The drug was injected within 15 min after its preparation (solubilized in sodium citrate buffer, pH 4.5).

T1D was induced in 10-week-old mice (*n* = 30) injected with an initial dose of 180 mg/kg STZ (Sigma-Aldrich, St. Louis, MO) followed by 100 mg/kg for another 2 consecutive days.

The control group (non-diabetic mice) was composed of 10-week-old mice (*n* = 10) injected with saline, following the same procedure for the diabetes-induced group. The onset of T1D/hyperglycemia was defined as blood glucose levels ≥11.1 mmol/l after 8 h of starvation. Restored hyperglycemia was defined as blood glucose concentrations <11.1 mmol/l for at least two consecutive weeks, without reverting to hyperglycemia on any subsequent day.

### Isolation, culture and characterization of hUCWJCs

The protocols for sampling human umbilical cord tissue were approved by the Institutional Review Board of Shantou University Medical College. All participants provided their written consent to participate in this study. The hUCWJC culture procedures were in accordance with those described by [23], with minor modifications. Briefly, human umbilical cords were obtained from consenting patients delivering full-term male infants by cesarean section at the Second Affiliated Hospital of Shantou University Medical College. The arteries and veins of each tissue were mechanically removed, and the subamnion region of the Wharton jelly was transferred to a sterile container and diced into small fragments. The explants were transferred into 100-mm plates with fresh growth media, comprising H-DMEM (Gibco) supplemented with 2% fetal bovine serum (FBS), basic Fibroblast Growth Factor (bFGF), transferrin, insulin, and selenium acid. The cells were left undisturbed for 7 days in a 37 °C humidified incubator with 5% CO_2_ to allow migration of cells from the explants. Thereafter, 4–5 ml of media was added every 2–3 days, and the cells were given sufficient time to reach near 100% confluence. Then, cells were digested with a 0.125% trypsin, 0.5 mM EDTA solution and replated at a 1:5 to 1:8 ratio.

The phenotypic properties of hUCWJCs were assessed on the basis of the immune response-related surface markers CD80, CD86, CD40, CD40L (data not shown), HLA-1 (MHC- I), and HLA-DR (MHC- II).

### Cell labeling

All the hUCWJCs in this work were labeled with 1,1′-dioctadecyl-3,3,3′,3′-tetramethylindocarbocyanine perchlorate (DiI), according to Xiong et al. [55] with minor modifications. Briefly, cells were harvested by treatment with 0.125% trypsin/0.5 mM EDTA, washed with DPBS and then resuspended and incubated for 30 min at 37 °C in darkness with growth medium containing 10 mM DiI. After washing with DPBS (1000 rpm, 5 min), hUCWJCs were immediately used for transplantation or the detection of labeling efficiency via phase contrast fluorescence microscopy.

### Cell transplantation

Diabetic mice with sustained hyperglycemia for at least 2 weeks were administered 1 × 10^7^ hUCWJCs (passage 5 to 6) via IP injection after 8 h of starvation and kept under the same conditions for another 4 h post- administration. hUCWJCs were infused in one single dose. To avoid cell aggregation, the cells were suspended in 500 μl of growth media and then injected into the right flank of the IP cavity. The same procedure was applied for the control group, but only growth media was injected.

### Immunohistochemistry and Immunofluorescence

Mouse paraffin-embedded tissue sections (4 to 6 μm thick) were dewaxed and then rehydrated through a graded series of ethanol to H_2_O. Antigen retrieval was performed using 10 mM citrate buffer (pH 6.0). The samples were blocked with 10% normal goat serum (Cell Signaling Technology, Danvers, MA 01923 USA) in DPBS at room temperature for 120 min and then, incubated overnight at 4 °C with the following primary antibodies: anti-Insulin (C27C9) rabbit mAb (1:400; Cell Signaling Technology, Danvers, MA 01923 USA); anti-glucagon (GCG) (D16G10) XP rabbit mAb (1:400; Cell Signaling Technology, Danvers, MA 01923 USA); or anti-PDX-1 (D59H3) XP rabbit mAb (1:400 Cell Signaling Technology, Danvers, MA 01923 USA). Samples were washed three times in DPBS for 5 min and then incubated at room temperature with goat anti-rabbit IgG (H + L), (ab’)_2_ fragment, Alexa Fluor 488 conjugate, 1:1000 (Cell Signaling Technology, Danvers, MA 01923 USA) or goat anti-rabbit IgG (H + L), (ab’)_2_ fragment, Alexa Fluor 594 conjugate, 1:1000 (Cell Signaling Technology, Danvers, MA 01923 USA) for 120 min. Nuclei were counterstained with 1,5-bis{[2-(di-methylamino) ethyl]amino}-4,8-dihydroxyanthracene-9,10-dione (DRAQ5), 1:1000 (Cell Signaling Technology, Danvers, MA 01923 USA). Images were acquired using a Fluoview FV1000 confocal laser scanning biological microscope (Olympus Corporation, Tokyo, Japan). DRAQ5, Alexa Fluor 488 conjugate and Alexa Fluor 594 conjugate were excited at 647, 488 and 594 nm, respectively. The area distribution of immunostained pancreatic islets was calculated with ImageJ software, and islets per section were counted manually.

### Assays for blood glucose and C-peptide

The glucose level in tail vein blood was monitored weekly, after 8 h of daylight fasting, with a glucometer OneTouch® UltraVue™ (Lifescan, Johnson&Johnson) [9].

C-peptide was assayed from the serum separated from blood collected via intracardiac puncture of anesthetized mice, using either human or mouse C-peptide enzyme-linked immunosorbent assay)(ELISA) kits (EMD Millipore, Missouri 63,394 USA). Optical Density (O.D.) was read at 450 nm with an Infinite® F200 (Tecan Trading AG) computer controlled plate reader. C-peptide was protected from proteolysis during storage and assay procedures by the addition of Trasylol (Aprotinin) at a concentration of 500 KIU/ml of serum.

### Quantitative real-time polymerase chain reaction (qPCR) assay

Tissues, previously frozen at −80 °C, were homogenized, and DNA was extracted with Takara MiniBEST Universal RNA Extraction Kit, Takara Bio. Inc. The complete list of primers is listed in Additional file 1: Table S1. The total DNA concentration and O.D. was assayed via UV spectrophotometry. Genomic DNA elimination was performed with Takara RR047Q. qPCR was performed with 200 ng of target DNA, ALU-specific primers with a hydrolysis probe (Sangon Biotech, Shanghai Co., Ltd.) (The amount of target DNA was corrected for the mouse albumin gene level to normalize genomic DNA values), human and mouse insulin-specific primers with a hydrolysis probe (Shanghai GenePharma Co., Ltd.) or SYBR Premix Ex Taq™ (Takara RR420Q) using an automated Model ABI7500 instrument (Applied Biosystems, Foster City, CA). Samples were run in triplicate.

### Urine assay

hUCWJC-treated and untreated diabetic mice were placed in individual metabolic cages (MMC100, Hatteras Instruments, Inc.) on day 26, and 24-h urine samples were collected to evaluate polyuria and polydipsia.

### Statistical analysis

The data were analyzed with the two tailed paired Student’s t-test and one-way ANOVA, and processed using Microsoft Excel 2010. *P* < 0.05 was considered statistically significant.

## Results

### Characterization of hUCWJCs

hUCWJCs remained as a monolayer on the culture plate during the sub-culturing process and were efficiently maintained in vitro with robust proliferation. Cells showed no signs of differentiation or senescence up to passage 6 (data not shown). Approximately 5 × 10^6^ to 8.5 × 10^6^ cells were cultured per 100-mm dish with a passage rate up to 1/8 and a doubling time of 19.3 h (± 2.6) ([44] http://doubling-time.com/compute.php) up to passage 5. The morphological differences between hUCWJCs cultured with DMEM/10% FBS and those used for IP administration can be seen in Additional file 2: Figure S1.

### Generation of the diabetic animal model

Repeated high doses of STZ for 3 consecutive days induced T1D-like disease in immunocompetent mice. Most of the animals developed polydipsia and polyuria within 10 days, while hyperglycemia (≥ 11.1 mmol/l) was achieved 2 to3 weeks after the first STZ dose.

### Blood glucose levels in diabetic mice after IP transplantation of hUCWJCs

**Fig. 1:**
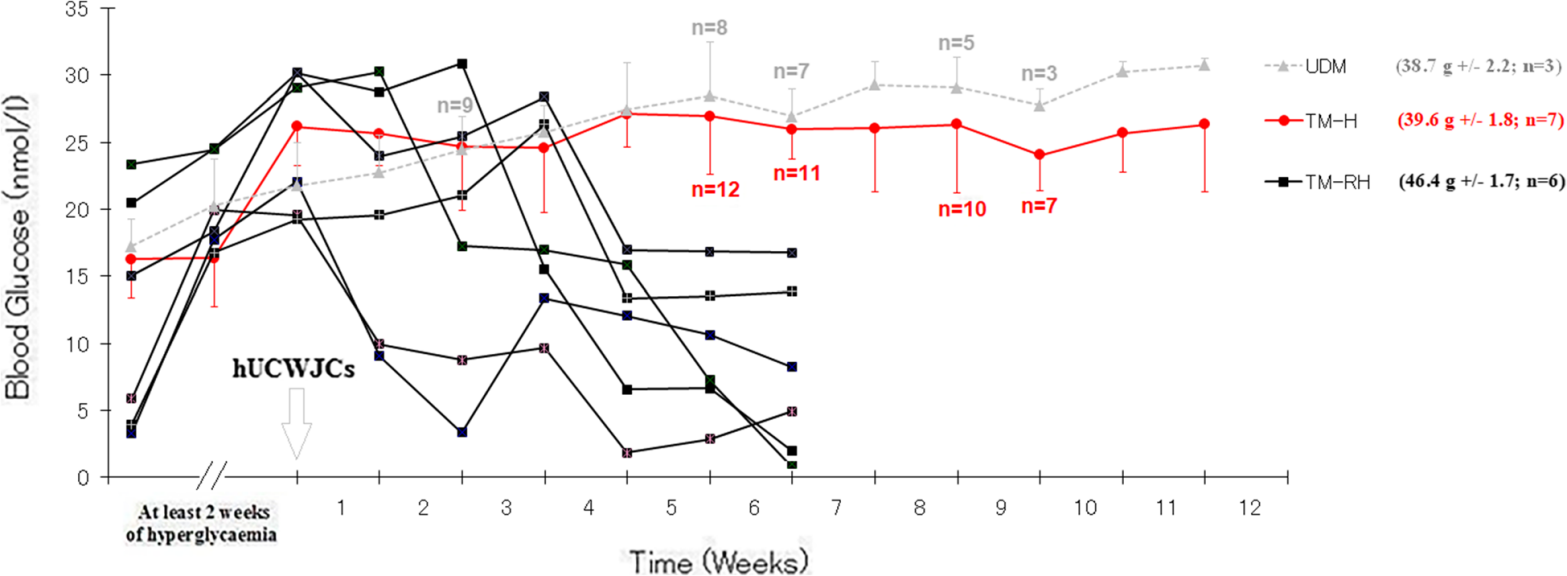
Blood glucose levels after hUCWJC infusion. (A) Blood glucose levels in TM. STZ was used to induce type I diabetes. Diabetic animals with hyperglycemia for at least 2 weeks were injected with 1 × 10^7^ hUCWJCs (passage 5/6) after 8 h of starvation. The grey dashed line represents the blood glucose levels in the control group, composed by untreated diabetic mice (UDM). The red line represents the TM-H (the data are the means ± s.d.), and the black lines are the TM-RH. The survival rate of the control group was 30% compared to 50% in TM-H. No significant differences in the body weight of the control group and TM-H were noticed at the end of the 11 weeks of monitoring (38.7 g +/− 2.2; *n* = 3 and 39.6 g +/− 1.8; *n* = 7 respectively). However the body weight of the two groups appeared to be significantly lower (*P < 0.05*) than that of the TM-RH at the time of sacrifice (46.4 g +/− 1.7; *n* = 6)

Six weeks after cell administration, 30% of the hUCWJC-treated mice (TM) showed restored hyperglycemia (TM-RH). These animals were then sacrificed and further analyzed. The remaining 70% of the TM continued to be hyperglycemic (TM-H) and were monitored for a total period of 11 weeks (Fig. 1). Hyperglycemia in the control group (untreated diabetic mice) progressively increased throughout the entire study period, and the survival rate was lower (*P* < 0.05) than that of TM-H (50% against 30%). Moreover, no differences in body weight between the control and TM-H groups were noticed at the end of the 11 weeks of monitoring (38.7 g +/− 2.2; *n* = 3 and 39.6 +/− 1.8; *n* = 7 respectively). Nonetheless the body weight of the two groups was significantly lower (*P < 0.05*) than that of the TM-RH at the time of sacrifice (46.4 g +/− 1.7; *n* = 6).

### Detection of hUCWJCs and human DNA in TM

**Fig. 2:**
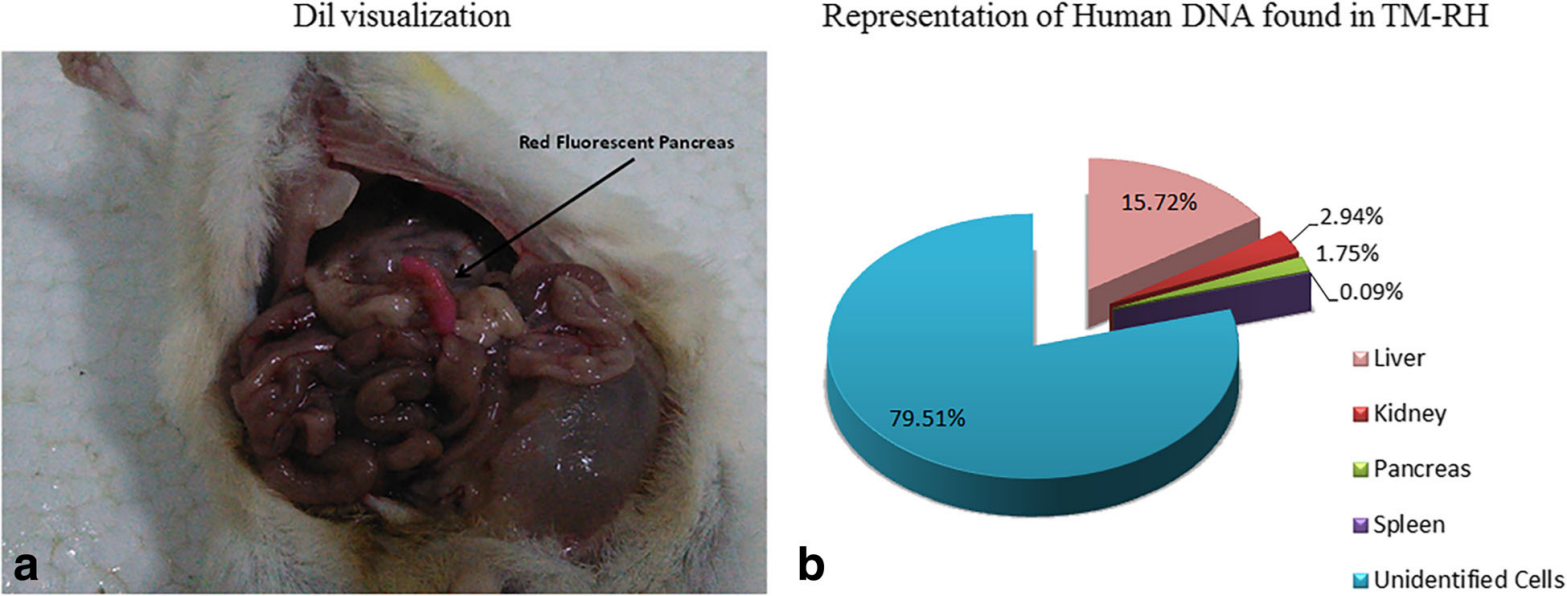
Detection of hUCWJCs via Dil detection and human ALU elements in TM. Red fluorescent regions in the pancreas were observed only in TM-H, 11 weeks after hUCWJC infusion (**a**). **b** A representative graphic of the distribution of human DNA in TM-RH. “Unidentified cells” are the percentage of human DNA not detected in the analyzed organs. The expression of human ALU sequences found in the TM was compared with that of 5 × 10^6^ hUCWJCs to calculate the # of cells engrafted in tissues. The data are the means ± s.d

The presence of hUCWJCs was confirmed by visualization of Dil with phase contrast fluorescence microscopy in the pancreas, kidney and liver of TM (data not shown). Interestingly, in some animals (4 out of 7 mice), the Dil-labeled hUCWJCs stained the pancreas (Fig. 2a). This is evidence that the infused SCs may target and migrate to damaged organs. However, none of the TM-RH showed signs of Dil-stain.

Both TM-RH and TM-H were assayed for engraftment using qPCR for human ALU elements. The heart, lungs, stomach, liver, kidneys, pancreas and spleen were analyzed. Human DNA was detected in the pancreas, kidneys, liver and spleen (Additional file 3: Table S2). The number of hUCWJCs found in mouse tissue was calculated by comparing the expression of human ALU sequences in mice with those in 5 × 10^6^ hUCWJCs. TM-RH (Fig. 2b) showed the highest number of human ALU sequences located in the liver (15.72% = 1.57 × 10^6^ ± 2.87 × 10^6^ s.d.), followed by the kidneys (2.94% = 2.94 × 10^5^ ± 1.16 × 10^5^ s.d.), and pancreas (1.75% = 1.75 × 10^5^ ± 0.75 × 10^5^ s.d.) and a few in the spleen (0.09% = 9 × 10^3^ ± 5 × 10^3^ s.d.).

TM-H were also positive for human ALU sequences in the pancreas, kidneys, liver and spleen. However, the expression in the different tissues varied considerably between animals, with values ranging from equal to those observed in TM-RH (Additional file 3: Table S2) to completely absent.

### Transplantation of hUCWJCs promoted insulin and mouse C-peptide formation

**Fig. 3:**
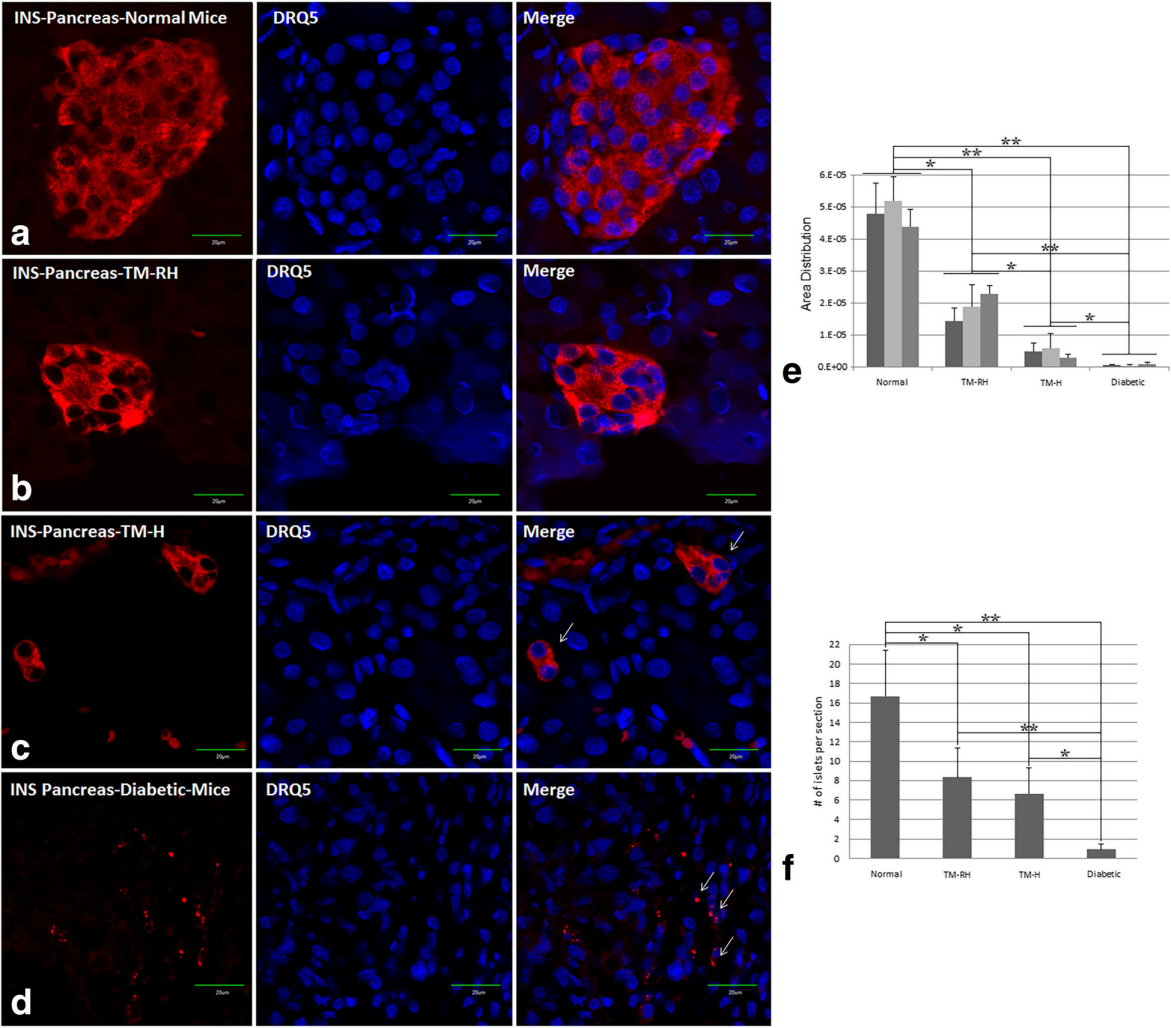
Immunofluorescence detection and insulin quantification. Representative pictures of pancreatic islets in the different groups. Immunofluorescence detection was performed with the primary anti-insulin (C27C9) rabbit mAb, 1:400 (mouse and human cross-reactivity). In contrast to islets from normal mice (**a**), pancreases from TM-RH (**b**), TM-H (**c**), and untreated diabetic mice (**d**) presented smaller insulin-producing islets (**e**). Moreover, the treated groups presented fewer islets per section than normal mice (**f**). Arrows in (**c**) and (**d**) indicate the localization of insulin formation. Different shades of gray bars in (**e**) represent different mice. INS scale bar = 20 μm. The data are the means ± s.d. *P < 0.05; **P < 0.01. Area distribution was calculated with ImageJ software. The islets per section were counted manually (*n* = 5)

An insulin analysis (mouse and human cross-reactivity) using confocal microscopy (Fig. 3) showed that unlike pancreatic islets from normal mice (Fig. 3a), TM-RH (Fig. 3b), TM-H (Fig. 3c) and untreated diabetic mice (Fig. 3d) presented smaller insulin-producing islets (Fig. 3e). Moreover, the number of islets per section in the TM was significantly lower than that in normal animals (Fig. 3f). Clearly defined clusters of insulin-producing cells were rare or absent in the untreated diabetic mice (Fig. 3d), confirming the toxicity of high doses of STZ to β-cell DNA.

**Fig. 4:**
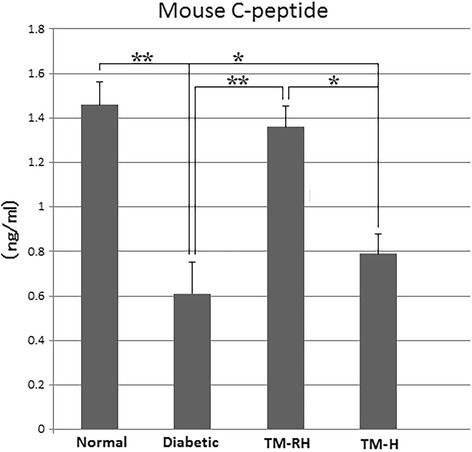
Analysis of mouse C-peptide. The analysis of mouse C-peptide was performed with a Millipore ELISA kit, and the Optical Density (O.D.) was read at 450 nm. IP administration of hUCWJCs into diabetic mice significantly increased the levels of mouse C-peptide in sera (TM-RH) compared to untreated diabetic mice and TM-H. The data are the means ± s.d. *P < 0.05; **P < 0.01

**Fig. 5:**
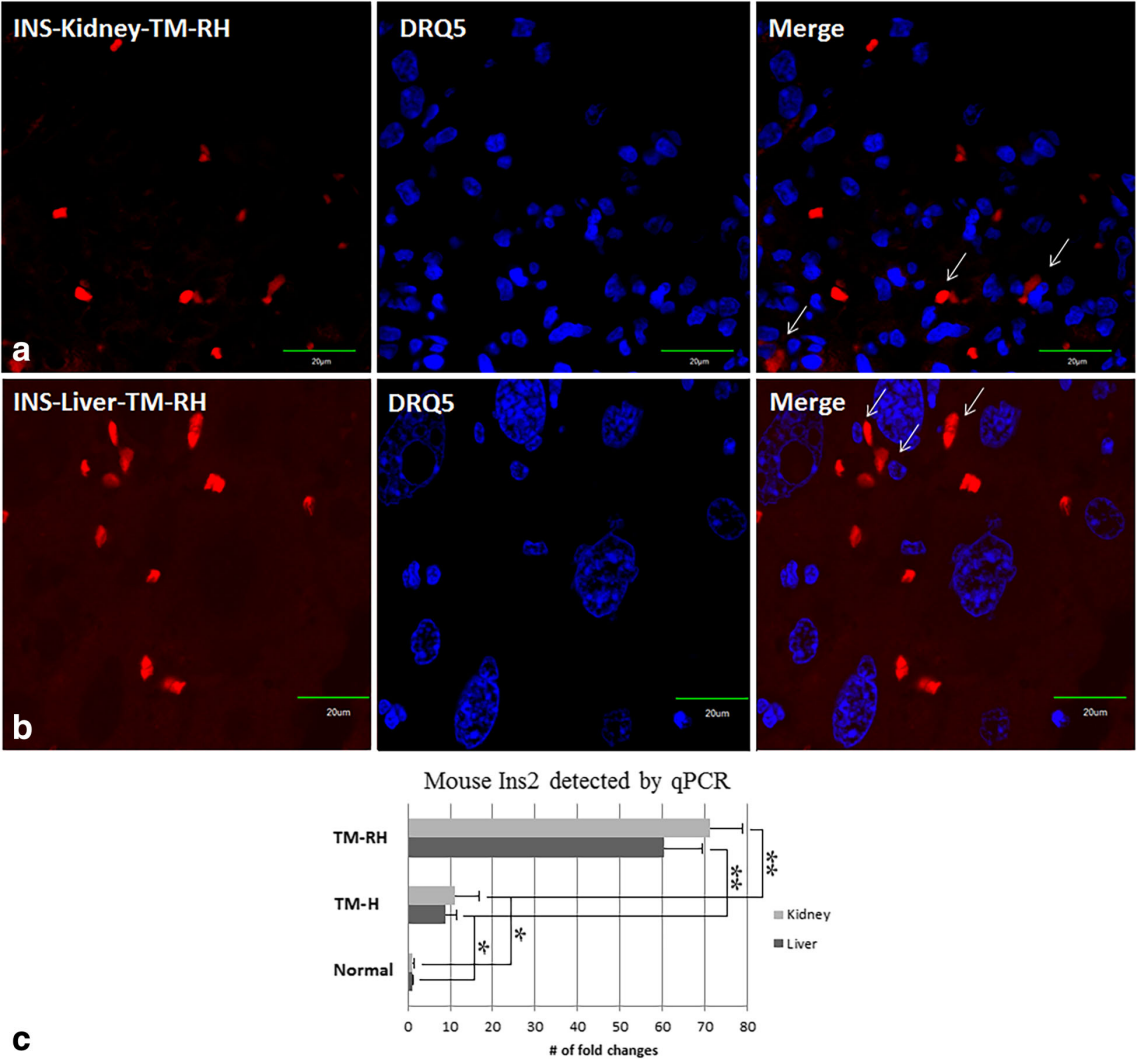
Insulin detection in the kidneys and liver using immunofluorescence and qPCR. Representative pictures of isolated insulin-producing cells found in the kidneys (**a**) and liver (**b**) of TM-RH using confocal microscopy. The mouse Ins2 gene level was analyzed using qPCR with a hydrolysis probe (**c**). Mouse Ins2 was found to be 60.4 (± 7.4) and 71.3 (± 8.2) fold higher in the liver and kidney, respectively, of TM-RH than in normal mice. The human INS transcript was not detected in the any of the transplanted mice. Scale bar = 20 μm. The data are the means ± s.d. *P < 0.05; **P < 0.01

ELISAs demonstrated that TM-RH had increased levels of mouse C-peptide in sera compared to untreated mice (Fig. 4). Paradoxically, the levels of mouse C-peptide between these two groups were similar, and in disagreement with the amount and size of insulin islets found in the pancreases of the former group (Fig. 3e, f). Nonetheless, when the kidneys and liver were verified by confocal microscopy, the presence of isolated insulin-producing cells was detected (Fig. 5a, b). To determine whether this insulin was produced by the human transplanted or the resident cells, we measured human INS and mouse Ins2 (the homologous mouse gene of human insulin) genes using qPCR. Human INS was undetectable in any of the TM; however, mouse Ins2, the most relevant marker of β-cell differentiation, was found to be 60.4 (± 7.4) and 71.3 (± 8.2) fold higher in the liver and kidney, respectively, of TM-RH than in normal mice (Fig. 5c). These findings led to the hypothesis that the insulin produced in the substitute organs was due to differentiation of the resident SCs into insulin-producing cells. Although mouse Ins2 in the liver and kidneys of TM-H was significantly lower (*P* < 0.01) than that in animals with restored hyperglycemia, it was still overexpressed compared to that in normal mice (*P* < 0.05).

Human C-peptide was also analyzed via ELISA. However, the results were negative in all the treated animals.

### Differentiation of hUCWJCs after transplantation

The gene expression analysis results, before and after transplantation, are summarized in Table 1. hUCWJCs were analyzed using qPCR for POUF51 gene expression, a marker critically important in the self-renewal of undifferentiated ESCs [6]; the human leucocyte antigen HLA-1 and the MHC class II cell surface receptor HLA-DRA are markers involved in the immunogenic properties of hUCWJCs. In agreement with previous reports [37, 53], our hUCWJCs were positive for POUF51 and HLA-1, and as expected, HLA-DRA, which is closely related to “graft versus host disease” was not expressed before cell administration. The data collection, 6 (TM-RH) or 11 (TM-H) weeks after transplantation, showed that POUF51 was down-regulated in all the animals analyzed, except in the livers of TM-RH and TM-H, which displayed Dil-stained regions in their pancreas. The down-regulation of POUF51 indicated that hUCWJCs lost their potency and became specialized. Moreover, HLA-1, which was highly expressed in hUCWJCs prior to administration, was down-regulated at the transcriptional level after transplantation.

**Table 1:** Gene expression Analysis

Oligo name	Type	Untreated-Diabetic Mice (n = 5)				Normal Mice (n = 5)				TM-RH (*n* = 4)				TM-H (n = 7)				Dil-SP (n = 4)	hUC-WJCs	Fluorescent Detection
		Pa	Ki	Li	Sp	Pa	Ki	Li	Sp	Pa	Ki	Li	Sp	Pa	Ki	Li	Sp	Pa	BT	
Beta-Actin ^a^		+	+	+	+	+	+	+	+	+	+	+	+	+	+	+	+	+	+	SYBR
Alu-Sequence										+	+	+	–	+	+	+	–	+	+	hydrolysis
POUF51	ES	x	x	x	x	x	x	x	x	x	x	–	x	x	x	x	x	–	+	SYBR
HLA-1	MSC	x	x	x	x	x	x	x	x	x	x	x	x	x	x	x	x	x	+	SYBR
HLA-DR	MSC	x	x	x	x	x	x	x	x	x	x	x	x	x	x	x	x	x	x	SYBR
SOX17	DE	x	x	x	x	x	x	x	x	x	x	x	x	x	x	x	x	x		SYBR
FOXA2	DE	x	x	x	x	x	x	x	x	x	x	x	x	x	x	x	x	x		SYBR
Hnf4-Alpha	PG					–	+	+	–	–	+	+	–					–		SYBR
HNF6	PF						–	1f	x	–	–	32f ± 9.1f	–							SYBR
PDX1	PF	x	x	x	x	x	x	x	x	x	x	x	x					x		SYBR
NKX6–1	PE	x	–	x	x	x	–	x		x	–	–	x					x		SYBR
Ngn3	PE	x	x	x	x	x	x	x	x	x	x	–	x					x		SYBR
NEUROD	PE					x	x	x	x	x	x	–	x					x		SYBR
PAX6	PE					x	x	x	x	x	x	+	x					x		SYBR
Ptf1a		x	x	x	x	x	x	x	x	x	x	x	x					x		SYBR
MafA		x	x	x	x	x	x	x	x	x	x	–	x					x		SYBR
VEGF-A						x	–	1f	x	x	–	50 ± 2.3f	–					x		SYBR
HES1						–	1f	1f	–	–	−500 ± 15f	−7 ± 1.5f	–					–		SYBR
Human-INS	EN	x	x	x	x	x	x	x	x	x	x	x	x	x	x	x	x	x	x	hydrolysis
Human Glucagon	EN	x	x	x	x	x	x	x	x	x	x	x	x					x		SYBR
Mouse-INS2	EN	–	x	x	x	1f	1f	1f	x	−9.8f ± 4.4f	71.3 ± 8.2f	60.4 ± 7.4f	x	x	–	–	x	x	x	hydrolysis

Legend: Gene expression analysis in normal, diabetic and TM using qPCR. (MSC) Mesenchymal Stem Cells; (ES) Embryonic Stem; (DE) Definitive Endoderm; (PG) Primitive Gut tube; (PF) Posterior Foregut; (PE) Pancreatic Endoderm; (EN) Endocrine Stage; (Pa) Pancreas; (Ki) Kidney; (Li) Liver; (Sp) Spleen; (Dil-SP) Dil-stained pancreases; (BT) Before Transplantation. The gene expression was defined as highly expressed (+) when the *quantification cycle* (C_q_) < 30; poorly expressed (−) when C_q_ ≥ 30 < 35; not expressed (X) when C_q_ ≥ 35 or when there was no transcript amplification; and () not assayed

Significant differences in the gene expression between groups are expressed as fold change values. For each gene expressed as fold change in the Normal Mice group, the sample was set to 1, and the samples for comparison were normalized to this level. Positive fold change values represent upregulation. Negative values represent downregulation. Fold changes are defined directly in terms of ratios

^a^Each marker was analyzed with SYBR Green fluorescent detection, and the transcript levels were normalized to those of the endogenous control β-actin. The data are the means ± s.d

To determine whether hUCWJCs differentiated toward the β-cell lineage after transplantation, tissues positive for human ALU elements were further assessed for specific markers according to the in vivo process of pancreatic organogenesis [11]: Definitive Endoderm (DE); Primitive Gut tube (PG); Posterior Foregut (PF); Pancreatic Endoderm/Endocrine Precursor (PE); and Endocrine Stage (EN). The gene expression analysis results are also summarized in Table 1. To group and organize the qPCR results, we defined the gene expression as highly expressed (+) when the *quantification cycle* (C_q_) < 30; poorly expressed (−) when C_q_ ≥ 30 < 35; not expressed (X) when C_q_ ≥ 35 or when there was no transcript amplification; and () when not assayed. The expression of each marker was analyzed with SYBR Green fluorescence detection, and the transcript levels were normalized to the endogenous control β-actin (ACTB). Samples were run in triplicate, and data are expressed as the means ± s.d.

In this work, we found that the transcription factors SOX17 and FOXA2, which are involved in earlier stages of DE commitment [11], were not expressed in any of the analyzed groups.

Hepatocyte nuclear factor HNF4A, which is critical for liver development and expressed in pancreatic β-cells, remained undetectable in untreated diabetic mice and TM-H. In normal mice and TM-RH, HNF4A was detectable in the pancreas and spleen and highly expressed in the kidney and liver. Moreover, no significant differences were observed between normal mice and TM-RH.

HNF-6 has been reported to control pancreatic endocrine differentiation at the precursor stage [25] and was found to be 32 ± 9.1-fold higher in the livers of TM-RH than in livers of control group mice. NKX6–1, which plays a critical role in the control of insulin biosynthesis, insulin secretion, and β-cell proliferation [51]; NEUROG3, a transcription factor required for the differentiation of all endocrine cells [15]; and NEUROD1, a transactivator of the insulin gene critical for development of the endocrine pancreas [16] were upregulated in the livers of TM-RH. The paired box protein PAX6 was not detected in normal mice but was highly expressed in the liver and slightly expressed in the spleen of TM-RH. MAFA, a principal activator of islet β-cell formation [41] and a synergistic cooperator with NEUROD1 and PDX-1, was also expressed in the liver of TM-RH. Vascular endothelial growth factor-A (VEGF-A) has been reported to protect glomerular microvasculature in diabetes [47] and was not expressed in the pancreas of TM-RH but was expressed in the kidneys (−) and spleen, and VEGF-A was found to be 50 ± 2.3-fold higher in livers of TM-RH compared to those of normal mice.

Hairy and enhancer of split (HES1), which promotes cell replication and prevents cell differentiation [17], is a transcription factor that operates as a general negative regulator of endodermal endocrine differentiation [26, 48]. HES1 has been suggested to inhibit the pancreatic endocrine differentiation program in the biliary epithelium by repressing NEUROG3 expression [49]. In this work, HES1 was down-regulated by 500 ± 15-fold in the kidneys and 7 ± 1.5-fold in the liver of TM-RH compared to normal mice.

### hUCWJCs attenuated the renal damage induced by STZ

**Fig. 6:**
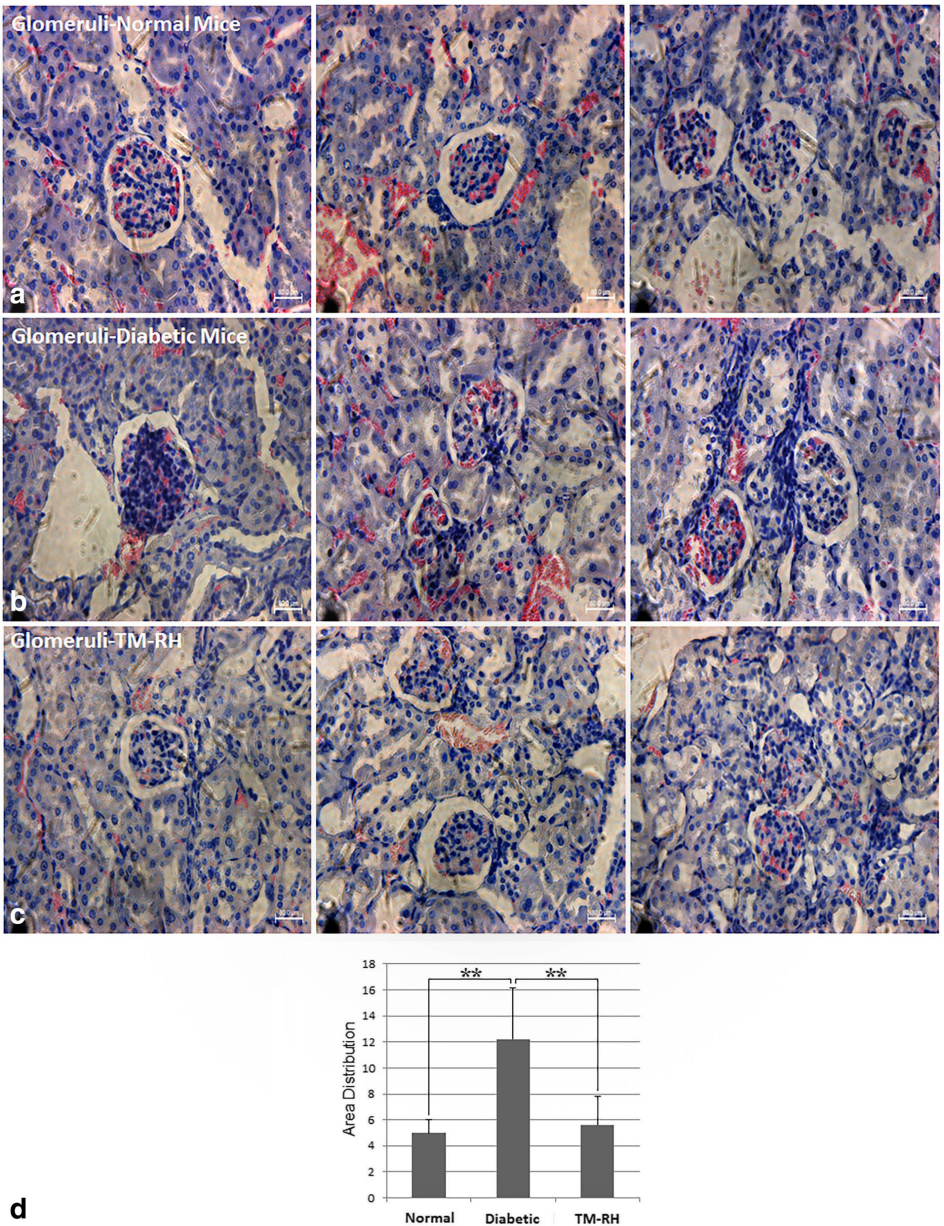
Glomerular morphology. Photomicrography of representative glomeruli from normal mice (**a**); untreated diabetic mice (**b**); and TM-RH (**c**). Glomeruli sections (5 μm) were stained with hematoxylin and eosin and magnified × 100. TM-RH showed attenuation of glomerular hypertrophy (**d**), with a morphology resembling glomeruli from normal mice, reduction in mesangial thickening and a decrease in the extracellular matrix protein deposits. Scale bar = 80 μm. The data are the means ± s.d. **P < 0.01. Area distribution was calculated with ImageJ software. Normal (n = 5); Diabetic (n = 5); TM-RH (n = 3). Glomeruli analyzed per animal (*n* = 15 to 20)

Overall, the glomeruli of TM-RH presented a morphology resembling that of normal mouse glomeruli (Fig. 6a, b, c), with attenuated mesangial thickening and visible signs of reduction in the extracellular matrix protein deposits. Moreover, TM-RH lacked glomerular hypertrophy (Fig. 6d).

Polydipsia (data not shown) and polyuria, a condition usually defined as excessive or abnormally large production or passage of urine, were assessed with individual metabolic cages for 24 h on day 28 after hUCWJC transplantation. The untreated diabetic mice had a marked increase in urinary volume compared to normal mice (9.11 ml ± 3.52 SD (*n* = 7) vs. 0.977 ml ± 0.38 SD (*n* = 5), *P* < 0.01). TM-RH showed a reduction in urinary volume compared to untreated diabetic animals (4.98 ml ± 2.43 SD (*n* = 3) vs. 9.11 ml ± 3.52 SD (n = 7), *P* < 0.05). Moreover, the excessive hunger or increased appetite produced by intracellular starvation that usually occurs early in the course of diabetic ketoacidosis [12] was also noticed in mice after STZ administration.

Six weeks after cell transplantation, the untreated diabetic mice weighed less than the TM-RH (39.07 g ± 2.01 SD vs. 42.35 g ± 1.35 SD; P *<* 0.05).

## Discussion

The transplanted human cells engrafted and survived in the damaged organs of diabetic mice for at least 11 weeks, in agreement with [55].

A study on the fate of intraperitoneally injected male rat bone marrow-derived stromal cells, tracked via Y-chromosome-sensitive PCR, reported that intraperitoneally injected Y-chromosome-positive cells were found in the heart muscle, lung, thymus, liver, spleen, kidney, skin, and femoral bone marrow of female Sprague Dawley rats [54]. In our study, hUCWJCs migrated and survived in the STZ damaged organs and nearby areas.

For the past few decades, several SC transplantation routes, such as IV [10], IC [34], through the orbital plexus [13] and IP [8] administration, have been implemented with varied results. For instance, IC administration appears to be advantageous over IV infusion because it eliminates the risk of capillary lung obstruction. However, the effectiveness and invasiveness of the abovementioned techniques for clinical treatments is debatable. For our particular purpose, one of the advantages of IP infusion, in addition to the simplicity of the technique, is that a large number of cells can be infused in a single dose.

The pathways by which the cells reached the affected organs were not analyzed in this study. However, we speculate that hUCWJCs migrated in a necrotactic manner mediated by chemoattractant molecules and/or through specific signals released from the necrotic or apoptotic cells of the damaged organs. We also hypothesize that human cells (including the unidentified cells shown in Fig. 2b) that managed to survive in the host recipient were absorbed by the mesenteric capillaries and/or simply adhered in the IP cavity and the surface of the target and intraperitoneal organs.

Whether serving in targeting or assisting roles, hUCWJCs are pivotal in tissue growth, metabolism, maturation and repair [27, 45]. They interact with host cells and influence the SC niche through differentiation and/or paracrine signaling mechanisms [20, 35].

The present work supports the idea that remote cell signaling from hUCWJCs appeared to induce the resident SCs (located in proximity and thus in a similar microenvironment) to specialize into insulin-producing cells.

A unique property of MSCs is the lack of expression of MHC class II and other surface markers, such as CD11b, CD14, CD31, CD34 and CD45. This results in failure of MSCs to initiate CD4+ T cell activation and likely explains how MSCs escape the normal process of alloantigen recognition [39]. We found that the MHC class.

I cell surface receptor HLA-1 was down-regulated in hUCWJCs after transplantation; a property that has been reported to confer relative or complete resistance to virus and cancer cells to the lytic effect of major histocompatibility complex class I HLA antigen-restricted cytotoxic T lymphocytes, thus providing immunological escape ([7, 32, 40]). We may deduce from our findings that a possible mechanism by which hUCWJCs exert their immunomodulatory properties is through down-regulation of the cell surface receptor HLA-1.

hUCWJCs improved the hyperglycemia symptoms in 30% of the TM. Kang et al. [28] reported that transplantation of wild-type bone marrow in NOD mice lowered the blood sugar of mice if the transplant was performed “before” but not after the onset of hyperglycemia. In this study, a significant number of hUCWJC-TM recovered from hyperglycemia even when the transplantation occurred at least 2 weeks after the onset of hyperglycemia. However, we speculate that our results are also correlated with the severity of hyperglycemia and organ damage. Moreover, because the majority of the TM (70%) did not positively respond to the SC treatment, our findings suggests that further experiments are needed to validate these claims and thus evaluate possible applications for clinical use.

We found that 4 out of 7 of the TM-H presented red fluorescence in their pancreas. Although the qPCR analysis showed that Dil-stained regions of the pancreases were rich in human DNA, it is possible that due to the uninhabitable conditions of the damaged organ, the infused cells were deceased and released the Dil, staining the targeted area. This is in agreement with the fact that red regions were not positive for human-specific markers of β-cell differentiation, and none of the TM-RH presented these fluorescence characteristics (Table 1).

The immunofluorescence analysis of insulin showed smaller and fewer insulin-producing clusters in pancreases of TM-RH compared to normal mice. However, no significant differences in the concentration of mouse C-peptide in sera were found, suggesting a compensatory mechanism that may have occurred through the contribution of insulin-producing cells found in the kidneys and liver of the TM-RH. Some of the animals not only reversed hyperglycemia after hUCWJC transplantation but also showed severe symptoms of hypoglycemia under starvation conditions (≤ 1.5 nmol/l), a result that was in agreement with findings from previous assays under the same experimental conditions (data not shown).

During the analysis of differentiation toward the β-cell lineage, the expression of human-specific PE markers found particularly in the liver of TM-RH suggests that some of the infused human cells were triggered toward an insulin-producing and islet cell fate. However, the qPCR findings showed that hUCWJCs did not express EN stage markers (no human INS was detected).

The immunofluorescence analysis using confocal microscopy revealed rare amounts of human PDX-1 in the kidneys and liver and GCG (mouse and human reactivity) in the kidneys of some of the TM-RH but not in TM-H or untreated diabetic mice (Additional file 4: Figure S2). Although adult β-cells express only the hormone insulin, it is important to note that some fetal β-cells are polyhormonal and express GCG and somatostatin in addition to insulin [21]. Moreover, unlike glucose-responsive adult β-cells, β-cells present during early development may not respond to glucose with increased insulin secretion [2, 18].

Up to 3.1% of the infused hUCWJCs were detected in the kidneys of TM-RH. The microscopic detection by tracking Dil confirmed that cells were present not only in glomeruli but were relatively evenly distributed along the tissue. The qPCR analysis demonstrated that VEGF-A, which has been reported to protect the glomerular microvasculature in diabetes [47], was upregulated in kidneys of TM-RH. Moreover, these mice lacked glomerular hypertrophy (Fig. 6d), confirming the anti-inflammatory properties of hUCWJCs.

In summary, the most remarkable findings in this work were the overexpression of mouse Ins2 in the liver and kidney and the increase in mouse C-peptide in sera of TM-RH. These results are supported by a study demonstrating that transplanted pancreatic islet cells survive in the liver, spleen and beneath the kidney capsule of C57BL/6 mice [42] and by other studies reporting the ability of liver cells, adipose tissue, spleen, bone marrow, and thymus to synthesize insulin ([14, 33]).

## Conclusion

We have confirmed the potential of IP administration of hUCWJCs to target STZ-damaged tissues, normalize hyperglycemia, promote insulin secretion from extra-pancreatic resident cells and improve renal damage typical of patients with diabetes mellitus. hUCWJC-mediated promotion of endogenous transdifferentiation of mouse renal and hepatic cells into functional insulin-producing cells still lacks biological evidence and in this sense is a phenomenon that needs to be thoroughly reviewed. Finally, we have verified that hUCWJCs, upon transplantation, down-regulate the cell surface receptor HLA-1, a property that has been reported to confer relative or complete resistance to virus and cancer cells to the lytic effect of major histocompatibility complex class I HLA antigen-restricted cytotoxic T lymphocytes, and thus provide immunological escape.

## Additional files

Additional file 1: Table S1. List of primers. Legend: ^a^ National Center for Biotechnology Information (www.ncbi.nlm.nih.gov/) accession numbers. (DOCX 17 kb)

Additional file 2: Figure S1. Morphological differences of the hUCWJCs. Legend: Morphological differences between the hUCWJCs used for transplantation into diabetic mice (A) cultured in H-DMEM, supplemented with 2% FBS, bFGF, transferrin, insulin, and selenium acid and (B) hUCWJCs cultured in DMEM/10% FBS. The small size of our hUCWJCs for transplantation (1/5th the size of the cells in B) allowed the culture of >5 × 10^6^ to ≤8.5 × 10^6^ cells per 100-mm dish with a passage rate up to 1/10. These cells showed a doubling time of 19.3 (± 2.6) up to passage 5. hUCWJCs for transplantation are 1. Scale bar = 200 μm. (TIFF 2356 kb)

Additional file 3: Table S2. Distribution of ALU elements in TM. Legend: ALU-specific primers were detected using qPCR with a hydrolysis probe. The values are expressed as the percentage of the total infused cells. “Unidentified cells” are the percentage of human DNA not detected in the analyzed organs. The number of hUCWJCs found in mouse tissue was calculated by comparing the expression of human ALU sequences in mice with those of 5 × 10^6^ hUCWJCs. qPCR samples were run in triplicate. (DOCX 16 kb)

Additional file 4: Figure S2. Title: Immunofluorescence assay of PDX-1 and glucagon. Legend: Immunofluorescence detection was performed with the following primary antibodies: anti-PDX1 (D59H3) XP rabbit mAb, 1:400 (human reactivity); and anti-GCG (D16G10) XP rabbit mAb, 1:400 (mouse and human cross-reactivity). PDX-1 was found in the kidney (A) and liver (B) and GCG was found in the kidneys (D) of TM-RH. However, the proteins were particularly scant and rare. We could not detect PDX-1 in the pancreas or GCG in the liver of any of the TM. PDX-1 and GCG scale bar = 40 μm. (TIFF 710 kb)

## Abbreviations

bFGF: basic Fibroblast Growth Factor
C_q_: *quantification* cycle
DE: Definitive Endoderm
Dil: 1,1′-dioctadecyl-3,3,3′,3′-tetramethylindocarbocyanine perchlorate
EN: Endocrine Stage
ESC: Embryonic Stem Cells
FBS: Fetal Bovine Serum
hUCWJCs: human Umbilical Cord Wharton Jelly Cells
IP: Intraperitoneal
MSCs: Mesenchymal Stem Cells
PE: Pancreatic Endoderm/Endocrine Precursor
PF: Posterior Foregut
PG: Primitive Gut tube
qPCR: quantitative Real-Time Polymerase Chain Reaction
T1D: Type 1 Diabetes
TM: hUCWJC-Treated Mice
TM-H: hUCWJC-Treated Mice with Hyperglycemia
TM-RH: hUCWJC-Treated Mice with Restored Hyperglycemia
