# Supplementary material for: Human umbilical cord Wharton jelly cells promote extra-pancreatic insulin formation and repair of renal damage in STZ-induced diabetic mice
Source: Cell Commun Signal. 2017 Oct 17;15:43. doi: 10.1186/s12964-017-0199-5 (PMC5645864; doi:10.1186/s12964-017-0199-5)
Supplement: Supplementary file 1 — List of primers. Legend: a National Center for Biotechnology Information (www.ncbi.nlm.nih.gov/) accession numbers. (DOCX 17 kb) [file 12964_2017_199_MOESM1_ESM.docx]

| Oligo name | Sequence Forward (5’ to 3’) | Sequence Reverse (5’ to 3’) | Product Size (bp) | Accession Number^a^ |
| --- | --- | --- | --- | --- |
| ACTB | CCTCATGAAGATCCTCACCGA | TTGCCAATGGTGATGACCTGG | 192 | XM_006715764 |
| Alu Sequence | CATGGTGAAACCCCGTCTCTA | GCCTCAGCCTCCCGAGTAG | 91 | NM_001308161 |
| HLA-1 | GCAGACACGGAATGTGAAGG | GTAGGCTCTCAACTGCTCCG | 292 | NM_002116 |
| HLA-DRA | TCTTGTCTGTTCTGCCTCACTC | TTCCAGGTTGGCTTTGTCC | 350 | NM_019111 |
| POUF51 | GTGTTCAGCCAAAAGACCATCT | GGCCTGCATGAGGGTTTCT | 156 | NM_001159542 |
| SOX17 | GCATGACTCCGGTGTGAATCT | TCACACGTCAGGATAGTTGCAGT | 103 | NM_022454 |
| FOXA2 | ATTGCTGGTCGTTTGTTGTG | TACGTGTTCATGCCGTTCAT | 187 | NM_021784 |
| HNF6 | TGTGGAAGTGGCTGCAGGA | TGTGAAGACCAACCTGGGCT | 252 | XM_011521484 |
| PDX1 | CGGAACTTTCTATTTAGGATGTGG | AAGATGTGAAGGTCATACTGGCTC | 131 | NM_000209 |
| NKX6-1 | ATCTTCTGGCCCGGAGTGA | CGCCAAGTATTTTGTTTGTTCG | 180 | XM_006714230 |
| NEUROG3 | GGCTGTGGGTGCTAAGGGTAAG | CAGGGAGAAGCAGAAGGAACAA | 104 | NM_020999 |
| NEUROD1 | ATTGCACCAGCCCTTCCTTTGAT | ACTCGGCGGACGGTTCGTGTTT | 90 | NM_002500 |
| PAX6 | CGAATTCTGCAGGTGTCCAA | ACAGACCCCCTCGGACAGTAAT | 207 | NM_001310159 |
| human-INS | CCTCTACCAGCTGGAGAACTACTG | GTTCAAGGGCTTTATTCCATCTCT | 100 | NM_001291897 |
| h-INS-TaqMan | FAM-AACTAGACGCAGCCCGCAGGC-BHQ1 | |  |  |
| Hnf4A | GAGATCCATGGTGTTCAAGGA | GTCAAGGATGCGTATGGACA | 109 | XM_005260407 |
| Ptf1A | CAGGCCCAGAAGGTCATC | GGGAGGGAGGCCATAATC | 78 | NM_178161 |
| GCG | GCTGCCAAGGAATTCATTGC | CTTCAACAATGGCGACCTCTTC | 79 | NM_002054 |
| MAFA | TGCAGCAGCGGCACATTC | CGCCAGCTTCTCGTATTTCTCCTTGT | 128 | NM_201589 |
| VEGFA | CCCTGATGAGATCGAGTACAT | CGGCTTGTCACATCTGCAAGT | 373 | NM_001287044 |
| HES1 | TCAACACGACACCGGATAAACC | GGTACTTCCCCAGCACACTTG | 270 | NM_005524 |
| Mouse-Ins2 | ACCCAGGCTTTTGTCAAGCA | TCCAGTGCCAAGGTCTGAAG | 185 | NM_001185084 |
| M-Ins2-probe | FAM-TGGCTTCTTCTACACACCCATGTCCC-BHQ1 | |  |  |
| Mouse Alb1 | GAA AAC CAG GCG ACT ATC TCC A | TGC ACA CTT CCT GGT CCT CA | 154 | NM_009654 |
