# Supplementary figures and images for: Human umbilical cord Wharton jelly cells promote extra-pancreatic insulin formation and repair of renal damage in STZ-induced diabetic mice
Source: Cell Commun Signal. 2017 Oct 17;15:43. doi: 10.1186/s12964-017-0199-5 (PMC5645864; doi:10.1186/s12964-017-0199-5)

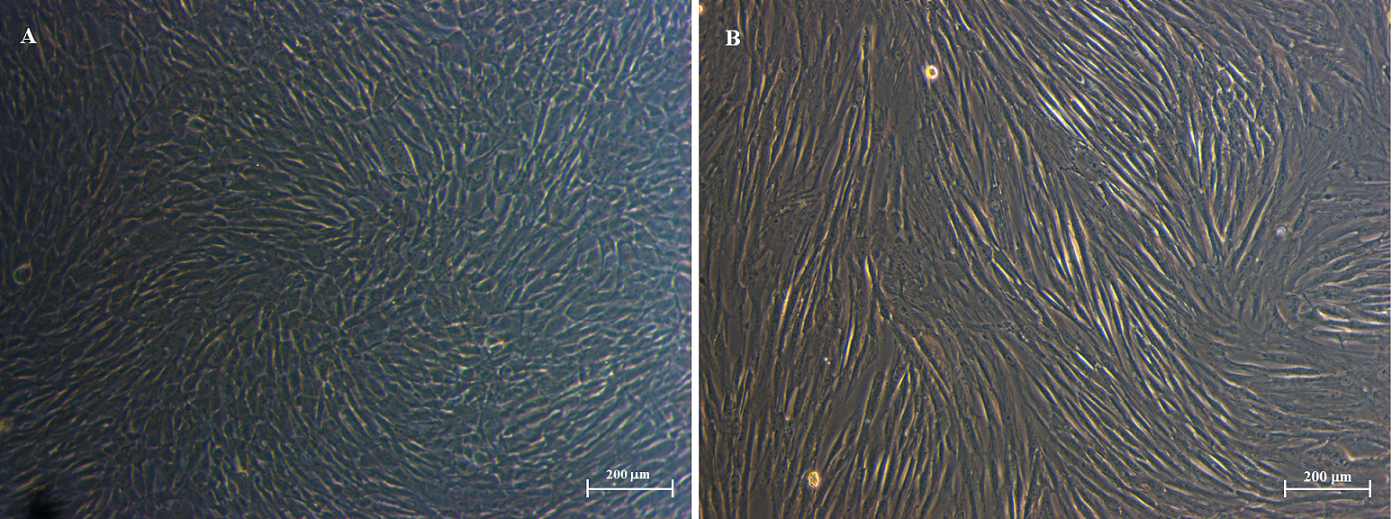

Supplement: Supplementary file 2 — Morphological differences of the hUCWJCs. Legend: Morphological differences between the hUCWJCs used for transplantation into diabetic mice (A) cultured in H-DMEM, supplemented with 2% FBS, bFGF, transferrin, insulin, and selenium acid and (B) hUCWJCs cultured in DMEM/10% FBS. The small size of our hUCWJCs for transplantation (1/5th the size of the cells in B) allowed the culture of >5 × 10^6^ to ≤8.5 × 10^6^ cells per 100-mm dish with a passage rate up to 1/10. These cells showed a doubling time of 19.3 (± 2.6) up to passage 5. hUCWJCs for transplantation are 1. Scale bar = 200 μm. (TIFF 2356 kb) [file 12964_2017_199_MOESM2_ESM.tif]

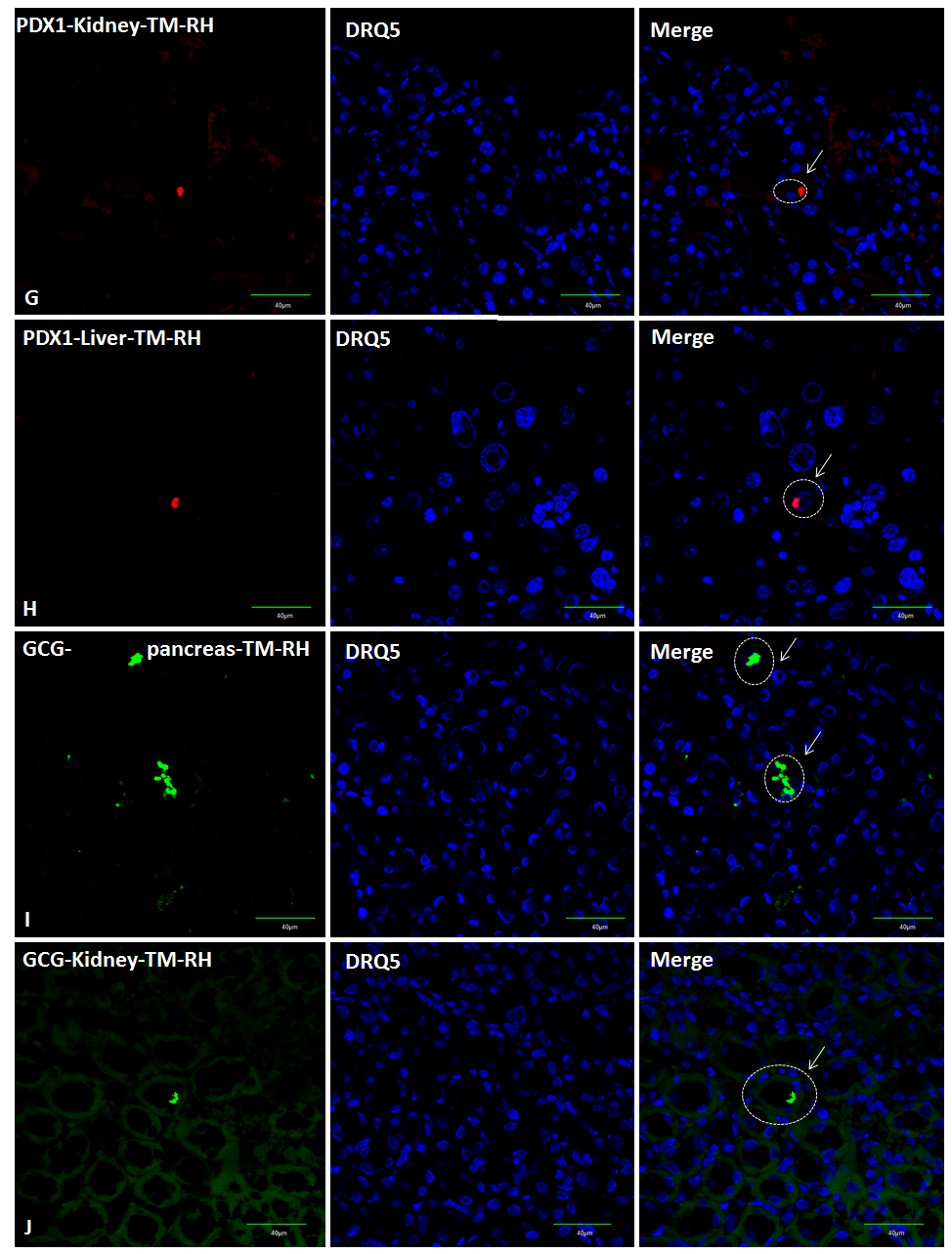

Supplement: Supplementary file 4 — Title: Immunofluorescence assay of PDX-1 and glucagon. Legend: Immunofluorescence detection was performed with the following primary antibodies: anti-PDX1 (D59H3) XP rabbit mAb, 1:400 (human reactivity); and anti-GCG (D16G10) XP rabbit mAb, 1:400 (mouse and human cross-reactivity). PDX-1 was found in the kidney (A) and liver (B) and GCG was found in the kidneys (D) of TM-RH. However, the proteins were particularly scant and rare. We could not detect PDX-1 in the pancreas or GCG in the liver of any of the TM. PDX-1 and GCG scale bar = 40 μm. (TIFF 710 kb) [file 12964_2017_199_MOESM4_ESM.tif]
