# Supplementary material for: Human umbilical cord Wharton jelly cells promote extra-pancreatic insulin formation and repair of renal damage in STZ-induced diabetic mice
Source: Cell Commun Signal. 2017 Oct 17;15:43. doi: 10.1186/s12964-017-0199-5 (PMC5645864; doi:10.1186/s12964-017-0199-5)
Supplement: Supplementary file 3 — Distribution of ALU elements in TM. Legend: ALU-specific primers were detected using qPCR with a hydrolysis probe. The values are expressed as the percentage of the total infused cells. “Unidentified cells” are the percentage of human DNA not detected in the analyzed organs. The number of hUCWJCs found in mouse tissue was calculated by comparing the expression of human ALU sequences in mice with those of 5 × 10^6^ hUCWJCs. qPCR samples were run in triplicate. (DOCX 16 kb) [file 12964_2017_199_MOESM3_ESM.docx]

| **Animal #** | **After treatment** | **Pancreas** | **Kidneys** | **Liver** | **Spleen** | **Unidentified cells** |
| --- | --- | --- | --- | --- | --- | --- |
| **TM-RH** |  |  |  |  |  |  |
| 1 | 6 weeks | 3.13 | 4.61 | 15.37 | 0.06 | 76.83 |
| 2 | 6 weeks | 0.96 | 2.04 | 12.76 | ND | 84.24 |
| 3 | 6 weeks | 1.69 | 2.96 | 17.96 | 0.09 | 77.3 |
| 4 | 6 weeks | 1.45 | 1.75 | 19.8 | 0.17 | 76.84 |
| 5 | 6 weeks | 1.37 | 2.23 | 12.45 | 0.04 | 83.91 |
| 6 | 6 weeks | 1.88 | 4.05 | 16.01 | 0.07 | 77.99 |
| **TM-H** |  |  |  |  |  |  |
| 1 | 6 weeks | 0.81 | 0.89 | 0.67 | ND | 97.63 |
| 2 | 6 weeks | 0.22 | 1.9 | 6.4 | 0.25 | 91.23 |
| 3 | 11 weeks | ND | 3.42 | 2.08 | 0.11 | 94.39 |
| 4 | 11 weeks | 1.83 | 1.17 | 2.21 | ND | 94.81 |
| 5 | 11 weeks | ND | 0.5 | 6.29 | ND | 93.2 |
| 6 | 11 weeks | 0.5 | 1 | 2.7 | 0.39 | 95.41 |
| 7 | 11 weeks | ND | ND | 0.8 | ND | 99.2 |
| 8 | 11 weeks | NA | NA | NA | NA | NA |
| 9 | 11 weeks | NA | NA | NA | NA | NA |

|  |  |  |
| --- | --- | --- |
|  |  |  |
